# Bee Venom Phospholipase A2, a Good “Chauffeur” for Delivering Tumor Antigen to the MHC I and MHC II Peptide-Loading Compartments of the Dendritic Cells: The Case of NY-ESO-1

**DOI:** 10.1371/journal.pone.0067645

**Published:** 2013-06-18

**Authors:** Christine Almunia, Marie Bretaudeau, Gerhard Held, Aurélie Babon, Charles Marchetti, Florence Anne Castelli, André Ménez, Bernard Maillere, Daniel Gillet

**Affiliations:** 1 Service d’Ingénierie Moléculaire des Protéines, Institut de Biologie et Technologies de Saclay, Commissariat à l'énergie atomique et aux énergies alternatives, Gif Sur Yvette, France; 2 Medizinische Klinik I, Universitaetsklinik des Saarlandes, Homburg, Germany; 3 Service de Biochimie et de Toxicologie nucléaire, Institut de Biologie Environnementale et Biotechnologie, Commissariat à l'énergie atomique et aux énergies alternatives, Bagnols sur Cèze, France; 4 Museum National d'Histoire Naturelle, Paris, France; University of Bergen, Norway

## Abstract

Bee venom phospholipase A2 (bvPLA2) is a small, 15kDa enzyme which hydrolyses many phospholipids through interfacial binding. The mutated bvPLA2H34Q (bvPLA2m), in which histidine-34 is replaced by glutamine, is not catalytically active. This protein has been shown to be a suitable membrane anchor and has been suggested as a suitable tumor-antigen vector for the development of novel dendritic cell-based vaccines. To confirm this feature, in this study the fusion protein PNY, composed of NY-ESO-1(NY(s)) fused to the C-terminus of bvPLA2m, was engineered. bvPLA2m enhanced the binding of NY(s) to the membrane of human monocyte-derived dendritic cells (DCs) and, once taken up by the cells, the antigen fused to the vector was directed to both MHC I and MHC II peptide-loading compartments. bvPLA2m was shown to increase the cross-presentation of the NY(s)-derived, restricted HLA-A*02 peptide, NY-ESO-1_157-165_(NY_157-165_), at the T1 cell surface. DCs loaded with the fusion protein induced cross-priming of NY(s)-specific CD8 + T-cells with greater efficiency than DCs loaded with NY(s). Sixty-five percent of these NY(s)-specific CD8+ T-cell lines could also be activated with the DCs pulsed with the peptide, NY_157-165_. Of these CD8+ T-cell lines, two were able to recognize the human melanoma cell line, SK-MEL-37, in a context of HLA-A*02. Only a small number of bvPLA2m CD8+ T-cell lines were induced, indicating the low immunogenicity of the protein. It was concluded that bvPLA2m can be used as a membrane-binding vector to promote MHC class II peptide presentation and MHC class I peptide cross-presentation. Such a system can, therefore, be tested for the preparation of cell-based vaccines.

## Introduction

Experimental vaccines, which have been studied primarily in the context of advanced cancers, have not, to date, been as successful as expected. For nearly two decades, much research and clinical development has focussed on the elaboration of new vaccine products, including viral, bacterial or yeast-based vaccines, protein or peptide-based vaccines, tumor-cell or tumor-cell-lysate-based vaccines and DNA- or RNA-based vaccines. Of these, only one, the sipuleucel-T (Provenge®) autologous vaccine, based on the use of DCs loaded with a recombinant fusion protein, has been approved by the FDA. Antigen (Ag)-pulsed dendritic cells (DCs) are one of the vaccine products emerging to treat cancers [1]. This immune therapy is used to modulate and boost the immune system to break down established tumor tolerance [2] and to fight the tumor expressing the target antigen. Dendritic cells are antigen-presenting cells (APC), and are the key element for activation of cells of the adaptive immune system through interaction between APC complexes (peptide-derived antigen/major histocompatibility complex (MHC)) and T-cell receptors (TcR), leading to T-cell activation. APCs hold both MHC class I and class II molecules which present peptide, respectively, to CD8+ cytotoxic T-cells, essential for the elimination of tumor cells, and to CD4+ T-cells, required to enhance and maintain the CD8+ T-cell response [3]. Thus, for complete T-cell activation and a productive immune response, cancer vaccines must be formulated with mature, antigen-pulsed DC(s), expressing the proper co-stimulatory molecules and bearing peptide-derived tumor protein on both MHC class I and class II molecules [4–6]. DCs pulsed with soluble, exogenous antigen preferentially stimulate CD4+ T-cells via MHC class II molecule/peptide complexes rather than by activation of CD8+ T-cells. The main source of MHC class I molecule-restricted peptides for stimulating CD8+ T-cells is proteasomic degradation of cytosolic protein [7]. Apart from the conventional presentation of epitopes derived from exogenous antigens on MHC class II molecules, DCs can also shuttle exogenous antigens to the MHC class I processing pathway for CD8+ T-cell activation in a special context [8,9]. This process, termed cross-presentation, plays a major role in immune defense against tumors. The challenge of defining the conditions and cellular context required for inducing a CD8+ T-cell response with antigen-pulsed dendritic cells has led to the design of a large number of vaccine strategies depending on peptide cross-presentation.

One of the major problems of cancer immunotherapy is poor antigen immunogenicity. Several vectors can be used to deliver recombinant proteins (costimulatory molecules, cytokines, growth factors, or genes expressing tumor-antigen targets) to antigen-presenting cells. The fusion protein, PA2024, included in the sipuleucel-T vaccine preparation, is composed of human prostatic acid phosphatase combined with granulocyte-macrophage colony-stimulating factor (GM-CSF). PA2024, internalized into DCs [10] via the GM-CSF receptor, was shown to be highly immunogenic and well tolerated, being derived from a consistent, well-defined manufacturing process that is scaleable. However, in clinical trials the vaccine was associated with a statistically significant survival benefit of only 4.5 months in men with metastatic prostate cancer [11,12]. Even if GM-CSF is an ideal adjuvant to stimulate an immune response and to augment tumor antigen presentation through the βc receptor [13], this stimulation is not strong enough to boost the immune system sufficiently to definitively eliminate prostate tumors. In the context of sipuleucel-T vaccine, antigen internalization was restricted to the GM-CSF receptor, limiting the amount of protein internalized. In contrast to GM-CSF, bvPLA2 interacts directly with cell membranes, enabling internalization of a large quantity of antigen. The protein binds tightly (Kd: 10^-12^ M) and irreversibly to the membrane through a combination of hydrophobic and electrostatic interactions with anionic phospholipids [14], leading to anchorage of the antigens fused to its C-terminus at the membrane cell surface, without cell-type distinction. bvPLA2 belongs to the secretory phospholipase A2 family whose members are active in many biological processes such as lipid metabolism, leading to possible cell toxicity. The bvPLA2 histidine-34 substitution with glutamine (H34Q) abolishes all of this biological activity. The mutant is thus a harmless protein without modified binding properties. The use of bee venom PLA2 (bvPLA2) for whole-Ag delivery is suggested here, as catalytically inactive bvPLA2H34Q [15] has been shown to induce cross-presentation of a peptide fused to it [16]. Hence, applied to antigen-presenting cells (APC), it is expected to prolong antigen presentation and cross-presentation on MHC molecules, thus enhancing CD4+ and CD8+ T-cell priming.

In this study, to demonstrate the ability of bvPLA2m to favor whole-antigen-derived peptide cross-presentation, NY-ESO-1 was chosen as the model antigen. The tumor antigen, NY-ESO-1, belongs to the category of “cancer/testis” antigens and is widely expressed in a number of cancers as well as in the normal testis [17,18]. Unlike other known cancer-associated antigens, a humoral and cellular immune response against NY-ESO-1 is frequently observed in patients with NY-ESO-1-expressing tumors [19,20]. It appears that peptides derived from NY-ESO-1 are capable of stimulating both CD4+ and CD8+ [21] lymphocytes via peptides presented on MHC class II and MHC class I molecules. It has been shown that NY-ESO-1 bound to calreticulin at the DC surface is internalized and reaches the MHC class II and MHC class I peptide-loading compartment [22]. Hence, as for the fusion protein in the sipuleucel-T vaccine, NY-ESO-1 internalization is receptor-mediated. However, the antigen is poorly cross-presented. Several restricted class I peptides derived from NY-ESO-1 have been described. One of these, the epitope NY_157-165_ restricted to HLA-A*02 molecules, is interesting to use as a marker of NY-ESO-1-derived peptide cross-presentation, as antibodies have been developed for detection of the MHC/peptide complex: HLA-A*02 /NY_157-165_ [23]. All of these features make NY-ESO-1 an ideal antigen to study the capacity of bvPLA2m to promote cross-presentation of its derived peptides.

To demonstrate the capacity of bvPLA2(m) to improve presentation and cross-presentation of NY-ESO-1(NY(s))-derived peptides to CD4+ and CD8+ T-cells, three DC vaccines were compared. The first, used as a control, was prepared with peptide-pulsed DCs, the second with NY(s)-loaded DCs and the third with DCs loaded with the fusion protein, PNY (NY(s) fused to the bvPLA2m C-terminus). Their efficacies to induce and activate NY(s)-specific lymphocytes were evaluated *in vitro*. Thereafter, cross-presentation efficiency was measured through NY_157-165_ presentation on HLA-A*02 molecules at the surface of T1 cells loaded with each protein. Protein cross-presentation levels and immunogenicity analyses demonstrated that using bvPLA2 increases NY-ESO-1 vaccine efficiency

## Results

### Design, expression and characterization of recombinant proteins

**Figure 1:**
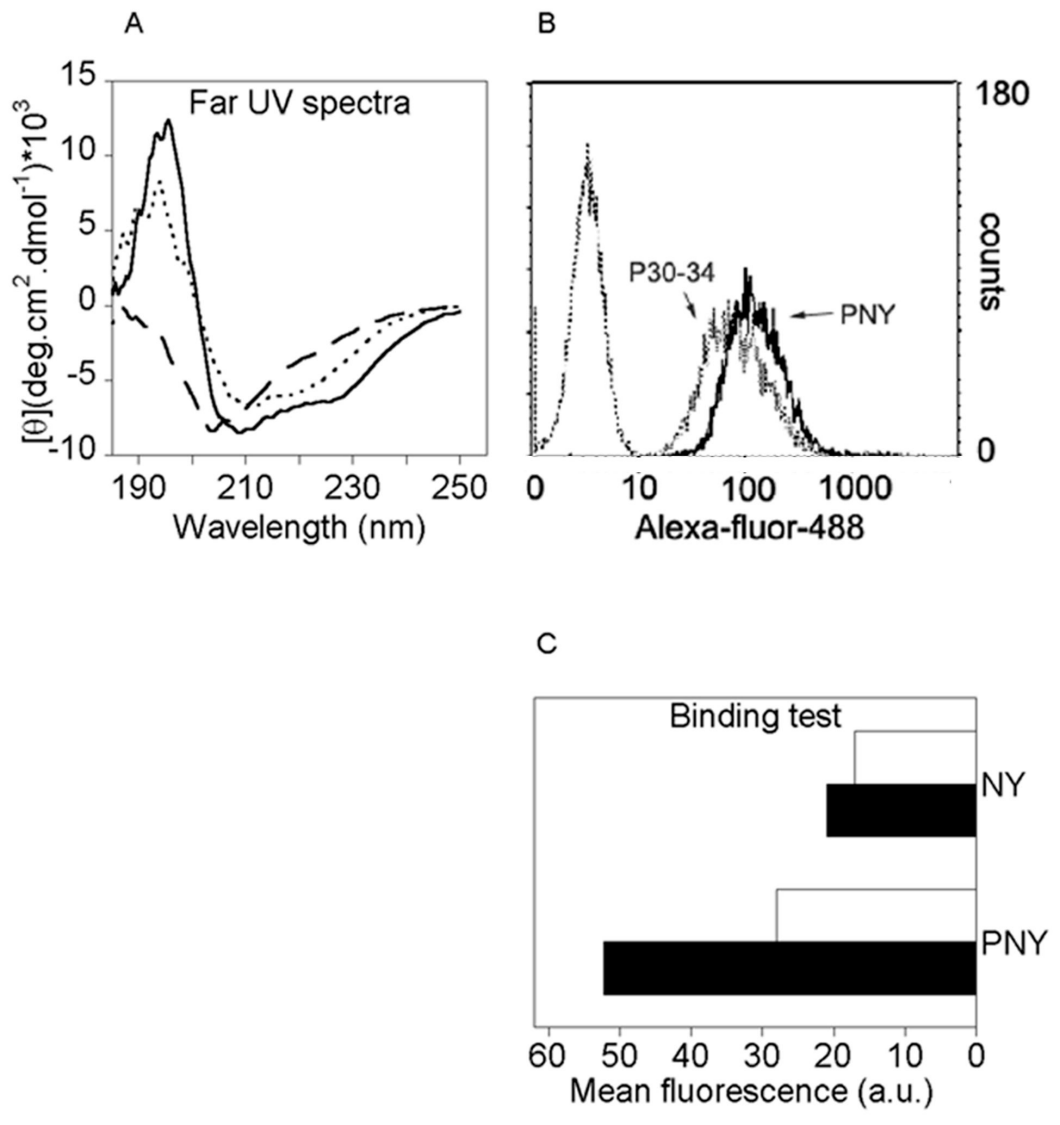
Fusion protein PNY secondary structure content and membrane binding analysis. A) Far-UV circular dichroiïsm spectra of recombinant bvPLA2m (solid), NY(s) (long dashes) and PNY (short dashes); B) FACS analysis of P30-34-Alexa-Fluor®488 (short dashes) and PNY-Alexa-Fluor®488 (solid) membrane binding; C) FACS analysis of NY(s)-Alexa-Fluor®488 and PNY-Alexa-Fluor®488 membrane binding, native form (■) and following denaturing treatment (□). Histogram represents the mean fluorescence of Alexa-Fluor® 488 normalized, taking into account each protein’s degree of labeling. DCs were incubated with proteins at 4 µM for 1.5 hours at 4°C.

Three recombinant proteins were produced in *E. coli*: bvPLA2 carrying the mutation H34Q, cancer/testis antigen NY-ESO-1 (referred to as NY(s)) and the fusion protein bvPLA2H34Q–NY-ESO-1 (referred to as PNY). To ensure strong binding to the membrane, bvPLA2m must be properly folded, especially its secondary structure, the α helix, which is involved in interfacial binding with membrane lipids. The secondary structure of each protein was investigated using far-UV CD spectroscopy. The spectra of bvPLA2 purified from bee venom and of recombinant bvPLA2m were similar (data not shown), corresponding to an approximate fraction of each secondary structure element calculated with K2D [24], 30% α helix, 12% β sheet and 57% random structure. This pattern was in agreement with the crystal structure of bvPLA2 [25]. K2D estimation of the secondary structure content of NY(s), suggested, with an accurate square distance, a proportion of 8% α helix, 42% β sheet and 50% random coil. The K2D analysis of the PNY spectrum indicated that the protein contains about 25% α helix, 25% β sheet and 50% random coil structure. The comparison between the spectra of recombinant proteins (Figure 1A) showed that the secondary structures of bvPLA2 are conserved in the fusion protein, probably resulting from the contribution of both fusion partners. Because the cell entry mechanism depends on protein size, the mean hydrodynamic diameter of each recombinant protein was measured by dynamic light scattering. The diameter was 140 nm for PNY, 103 nm for bvPLA2m and 44 nm for NY(s) in its soluble form. These results indicated that each protein was not aggregated and the fusion of NY(s) to bvPLA2m did not drastically change the protein size range. On the contrary, the mean hydrodynamic diameter of NY(a), was 313 nm, indicating its aggregation. For the latter, it could be expected that this will be phagocytozed.

### PNY anchorage to the DC surface and internalization pathway

**Figure 2:**
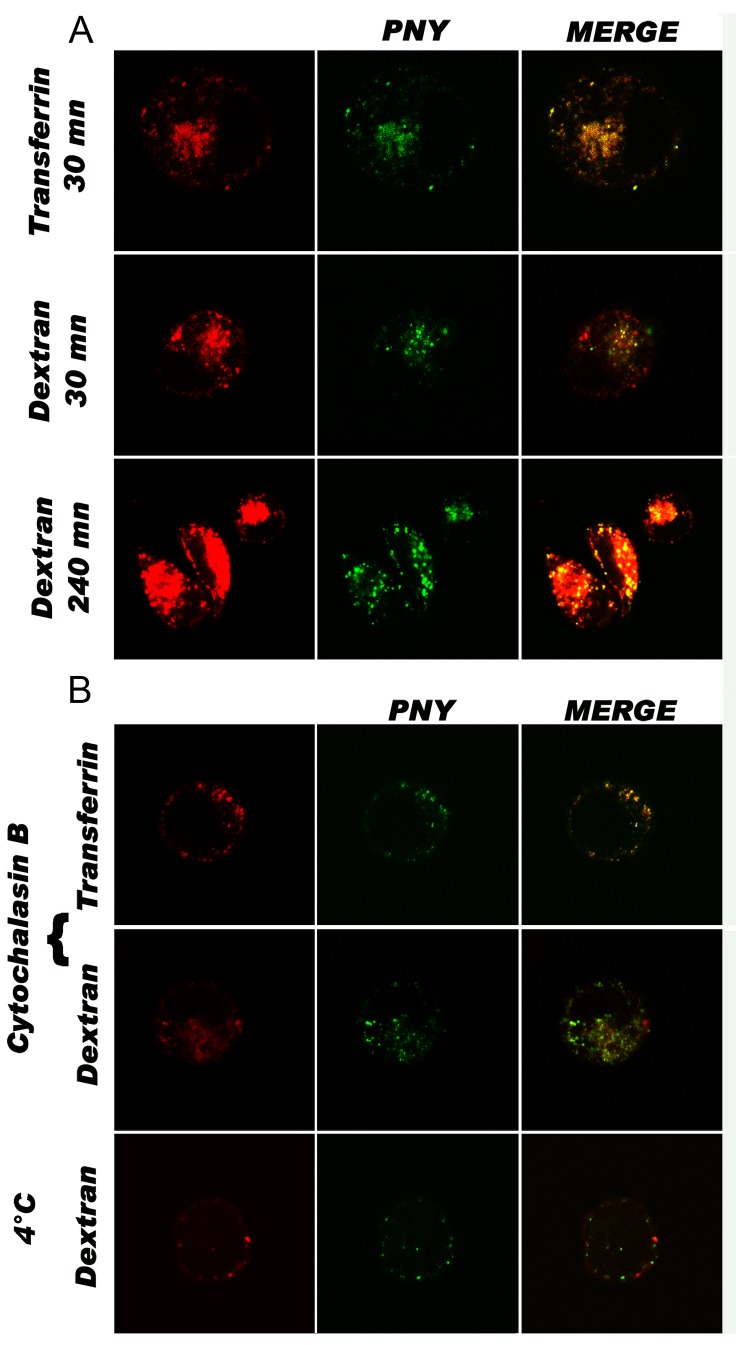
Internalization pathway and intracellular localization of PNY-Alexa-Fluor®488 into DCs. A) Immature monocyte-derived DCs preincubated with transferrin-TR (red) for 30 min and with PNY-Alexa-Fluor®488 (green) for 30 min at a final concentration of 3 µM (first line)? or incubated with dextran-TR (red) and PNY for 30 min (second line); DCs incubated with dextran and PNY for four hours (third line). B) Immature monocyte-derived DCs were pretreated with cytochalasin B for two hours before incubation with colocalization marker (red) and PNY-Alexa-Fluor®488 (green) (two upper lines); DCs incubated at 4°C with co-localization marker (red) and PNY-Alexa-Fluor®488 (green) (bottom line).

The anchorage and internalization pathway of bvPLA2 has already been characterized by Babon *et al*. [16]. In this study, the protein fused with a peptide at its C-terminus (P30-34) bound to the membrane efficiently and was transported via early endosomes to late endosomes and lysosomes. In the present study, the binding properties and intracellular pathway were verified to be the same as those characterized by Babon *et al.*, with a whole protein linked to bvPLA2. The two recombinant fusion proteins, P30-34 and PNY, showed similar binding efficiencies (Figure 1B). To evaluate the contribution of the bvPLA2m fraction of the fusion protein PNY, the binding efficiency of native PNY was compared with that of denatured PNY, and native (NY(s)) with denatured (NY(d)) NY (Figure 1C). Binding of native PNY to the membrane was more efficient than for unfolded PNY. The binding efficiency of unfolded PNY was quite similar to that of native NY(s) and denatured NY(d). This confirmed that PNY binding to the membrane is not modified by the fusion of NY(s) and that unfolded PNY binding is due to NY binding. Folded PNY binding to the membrane was at least twice that measured for soluble NY(s) to calreticulin. If protein internalization depends on membrane binding ability, it might be expected that peptide derived from PNY would be cross-presented at least twice as much as peptide derived from NY(s). P30-34 was also shown to be transported into the DCs, to late endo-lysosomes through early endosomes. Fusing NY(s) to bvPLA2 did not change its intracellular pathway. In fact, using intracellular, specific colocalization markers, it was illustrated by confocal microscopy that PNY colocalized with transferrin-Texas Red, a marker of early endosomes (Figure 2A) [26] and was found to be accumulated in the late endosomes stained with dextran-Texas Red. The colocalization analysis performed with jACoP [27] determined that a fraction of 91% ± 7% of PNY was superimposed with transferrin and 55% ± 7% overlapped with dextran in the first 90 min of incubation. After 4 h of incubation, almost of all the internalized PNY colocalized with dextran and transferrin. Intensity correlation quotient (ICQ) values, calculated from triplicate images, were 0.035 ± 0.020 and 0.037±0.08, respectively. ICQ values indicate a dependent staining when they are in the range of 0 to 0.5 [28]. Lowering the incubation temperature, which is known to stop fusion vesicles [29], prevented nearly all PNY internalization and intracellular traffic, indicating that fusion protein trafficking is vesicular (Figure 2B). In addition, PNY internalization was not fully inhibited by cytochalasin B which blocks both phagocytosis and macropinocytosis (Figure 2B).

### In vitro induction of NY-ESO-1-specific CD4+ T-cells

**Figure 3:**
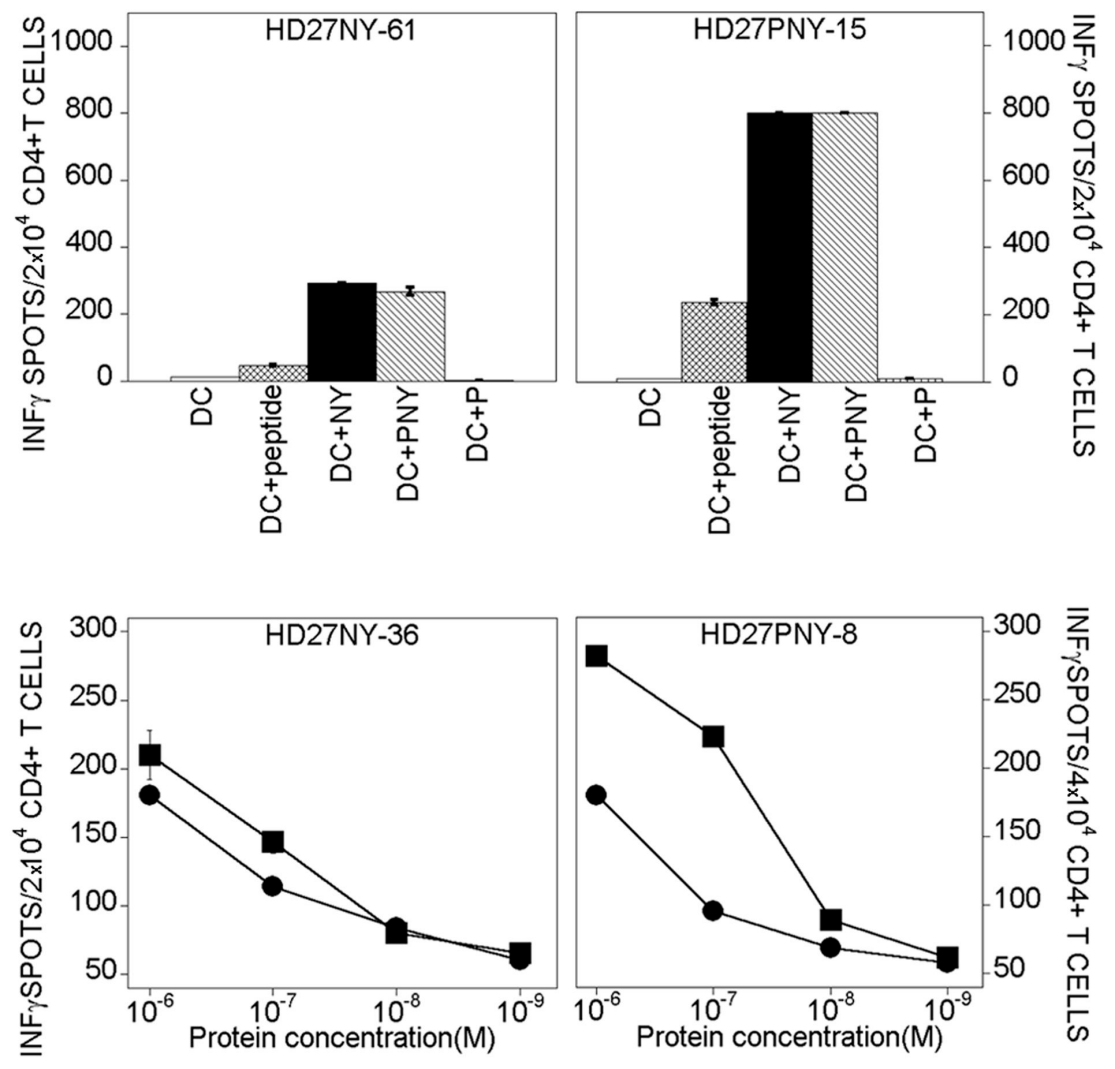
IFN-γ ELISPOT of CD4+ human T-cells induced with DCs loaded with either NY-ESO-1 or PNY. Upper line) Recognition of autologous DCs pulsed with NY-ESO-1_87-111_ or loaded with either NY(s) or PNY, by specific CD4+ T-cells induced with NY(s)-or PNY-loaded DCs at 3 µM. DC= DC non-loaded; DC+ peptide = DC+ NY-ESO-1_87-111_; DC+P = DC+ bvPLA2m. Bottom line) Dose-dependence of CD4+ T-cell-mediated recognition of NY(s). Target cells were DCs loaded with either NY(s) (●) or PNY (■). The cell lines were induced with either NY(s) or PNY at 3µM. Names of T-cell lines: Buffy coat preparation number + protein used to induce T-cell line + number of T-cell lines; HD27NY-61 and HD27NY-36 were generated with DCs loaded with NY(s); HD27PNY-15 and HD27PNY-8 were generated with DCs loaded with PNY.

In a series of *in vitro* experiments, CD4+ T-cells from DRB3*0101 healthy human donors were primed against NY(s) and PNY. Eighteen specific NY(s) CD4+ T-cell lines were generated from 7×10^6^ CD4 + T-cells stimulated with DCs loaded with PNY, whereas eleven specific NY(s) CD4+ T-cell lines were obtained with DCs loaded with NY. Surprisingly, none of the PNY-specific T-cell lines were activated with DCs loaded with bvPLA2m. Eight of each of the NY(s)-and PNY-specific T-cell lines were further characterized with DCs loaded with the MHC II-restricted peptide epitope, NY-ESO-1_87-111_ (Figure 3A). This peptide, released from the NY-ESO-1 degradation, was shown by Zarour *et al*. to be a highly immunogenic epitope forming a stable complex with MHC class II DRB3*0101 molecules [30]. The CD4+ T-cells were strongly activated, indicating that protein processing and presentation are the same as for NY-ESO-1.

Titration of the reactivity of these NY(s)-and PNY-specific CD4+ T-cells to NY(s) and PNY autologous presenting cells was evaluated in a 20-h IFN-γ ELISPOT. For the T-cell line, HD27NY-36, dose/response curves obtained with NY(s)-or PNY-loaded DCs were almost overlaid. In contrast, the T-cell line, HD27PNY-8, had a higher sensitivity to DCs loaded with PNY. This result might indicate either a higher presentation of peptide derived from PNY or that the activated T-cells were not monoclonal. Overall, presentation of peptides derived from NY(s) and PNY molecules occurred on MHC class II with the same efficiency and their specificity was the same as for the cancer testis antigen, NY-ESO-I.

### In vitro induction of NY-specific CD8+ T-cells

To investigate the capacity of PNY-loaded DCs to prime NY(s)-specific CD8+ T-cells, series of *in vitro* T-cell inductions were carried out, comparing DCs loaded with either PNY, NY(s) or NY_157-165_.

**Figure 4:**
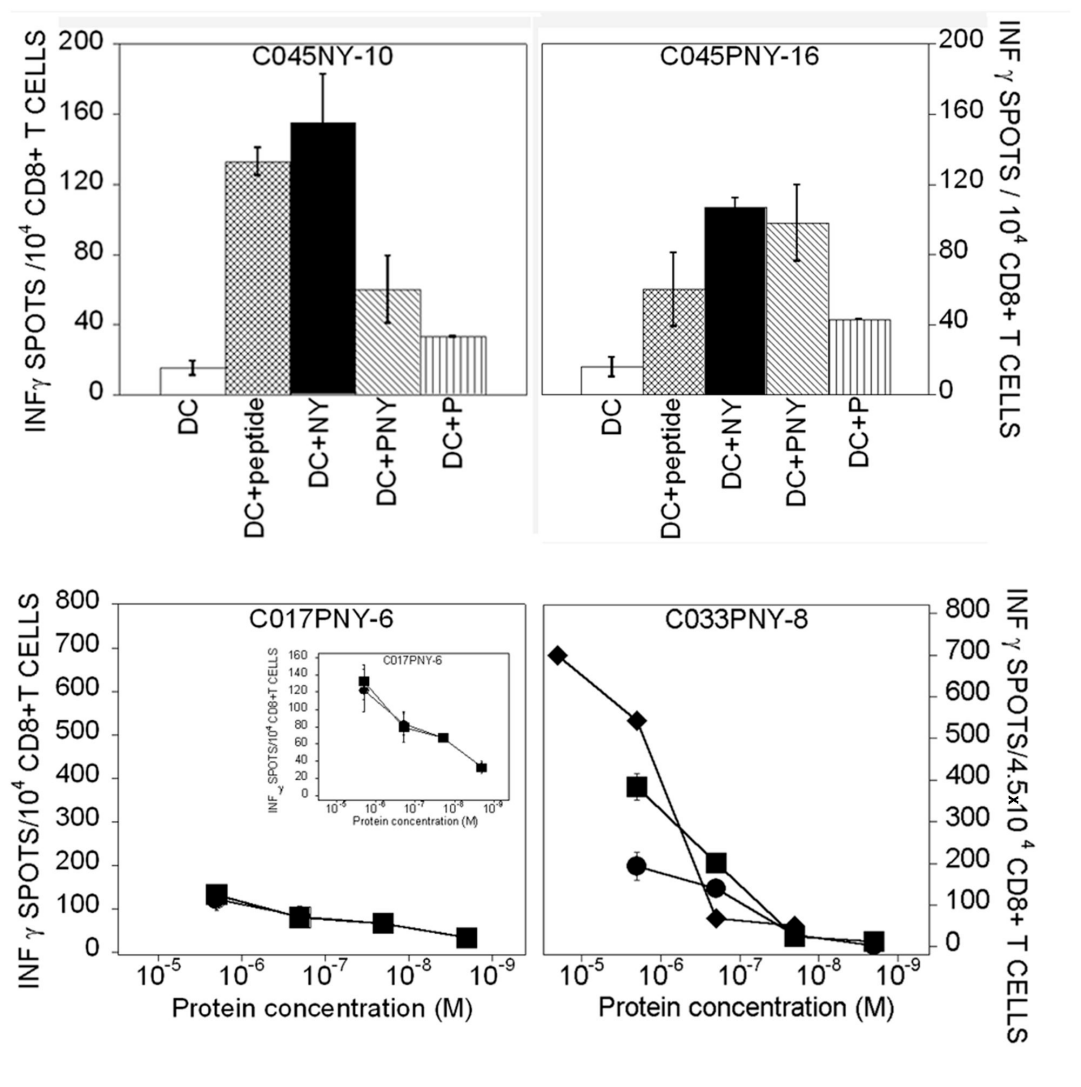
IFN-γ ELISPOT of CD8+ human T-cells induced with DCs loaded with either NY(s) or PNY. Upper line) Recognition of autologous DCs, pulsed with NY_157-165_ peptide or loaded with either NY(s) or PNY, by CD8+ T-cells induced with either NY(s)-or PNY-loaded DCs. Bottom line) Dose-dependence of CD8+ T-cell-mediated recognition of NY(s) or NY_157-165_ peptide. Target cells were DCs loaded with either NY(s) (●) or PNY (■) or pulsed with NY_157-165_ peptide (♦). The cell lines were induced with either NY(s) or PNY at 3µM. Names of T-cell lines: Buffy coat preparation number + protein used to induce T-cell line + number of T-cell line; C045NY-10 was generated with DCs loaded with NY(s); C045PNY-6, C033PNY-8 and C045PNY-16 were generated with DCs loaded with PNY.

The results described in Table 1 clearly demonstrate that PNY is more immunogenic than NY(s), and equivalently immunogenic to NY_157-165_. Surprisingly, from NY_157-165_-induced CD8+ T-cell lines, only three responded to DCs loaded with NY(s) or PNY (two of these are shown in Figure 4A) whereas the CD8+ T-cell lines primed with DCs loaded with whole protein were able to recognize NY_157-165_ at the DC surface (Figure 4A). The question arises concerning TcR binding constants, and their specificity with respect to CD8+ T-cells generated with peptide-pulsed APC for MHC/peptide complexes presented by tumor cells. Furthermore, PNY loaded DCs generated only three CD8+ T-cell lines specific for bvPLA2m, suggesting its low immunogenicity (Table 1).

**Table 1:** CD8+ T-cells lines generated with monocyte-derived-DCs loaded with NY_157-165_ peptide or PNY or NY(s) recombinant protein.

	Immunogen	CD8+ T-cell lines generated	**NY_157-165_** recognition		**NY(s)** recognition		**bvPLA2m** recognition		**Tumor cell line** recognition SK-MEL 37	
			Nb	%	Nb	%	Nb	%	Nb	%
	**NY_157-165_**	17	17	100	3	18	0	0	2	12
	**NY(s)**	6	5	84	6	100	0	0	0	0
	**PNY**	17	11	70	14	82	3	18	2	12
**Total**		**40**	**33**		**23**		**3**		**4**	

T-cell line specificity was characterized with autologous, immature DCs loaded with the peptide, NY_157-165_ (10 µM), or NY(s) or PNY recombinant proteins, or bvPLA2m (3µM). The results are from the *in vitro* generation of CD8+ T-cells from four healthy donors. Nb=Number of specific CD8+ T-cell lines.

Two uncloned CD8+ T-cell lines, C033PNY-8 and C017PNY-6, induced with PNY-loaded DCs, were used to evaluate the cross-presentation efficiency of DCs loaded with each protein (PNY and NY(s)). In the case of CO33PNY-6, the dose/response curves with each protein-loaded DCs are superimposed (Figure 4B). In contrast, for the CD8+ T-cell line, C017PNY-8, activation is higher with peptide-pulsed DCs than with protein-loaded DCs. Here, it can be hypothesized that, contrary to protein loading of DCs, peptide pulsing provides more MHC/peptide complexes, resulting in more TcR–NY_157-165_/MHC interaction. To conclude, NY(s)-and PNY-loaded DCs activated NY(s)-specific T-cell lines with at least the same efficiency.

To sum up, fusion of bvPLA2m to NY(s) enhanced immunogenicity but not the antigenicity of cancer/testis antigen. It can be supposed that the peptide generation kinetics from PNY, being slower than NY(s), could explain this result. To answer this question, PNY and NY(s) cross-presentation pathways were characterized and antigen cross-presentation kinetics were analyzed by detecting MHC molecules/NY_157-165_ at the DC surface.

### Intracellular cross-presentation pathway of PNY

Processing and cross-presentation pathways of a peptide fused to bvPLA2 were previously analyzed by Babon and colleagues [16]. Its presentation was demonstrated to be dependent on MHC class I molecule neo-synthesis without TAP and endosomal protease involvement. Here, the same experiments were carried out to verify that fusing a whole antigen would not modify the cross-presentation pathway. Four CD8+ T-cell lines were used to evaluate lymphocyte activation by DCs loaded with each protein and treated with inhibitors: i) C051PNY-15, an uncloned cell line induced with PNY-loaded DCs, and specific for both NY(s) and bvPLA2m; ii) C051PNY-4, a cell line induced with DCs loaded with PNY and specific for NY(s); iii) C051PEP-17, a monoclonal cell line induced with NY_157-165_-pulsed DCs; and iv) C040PNY-1, an uncloned cell line induced with bvPLA2m-loaded dendritic cells.

The DCs treated with inhibitors and pulsed with peptide NY_157-165_ were used as controls. The inhibition efficiency was evaluated using the following formula:

$$I=\frac{\text{Average nb}\text{.of spots from N}\text{.T}\text{.DCs}-\text{Average nb}\text{.of spots from T}\text{.DCs}}{\text{Average nb}\text{.of spots from N}\text{.T}\text{.DCs}}x100$$

I: inhibition efficiency
T. DCs: treated DCs
N.T.DCs: non-treated DCs

**Figure 5:**
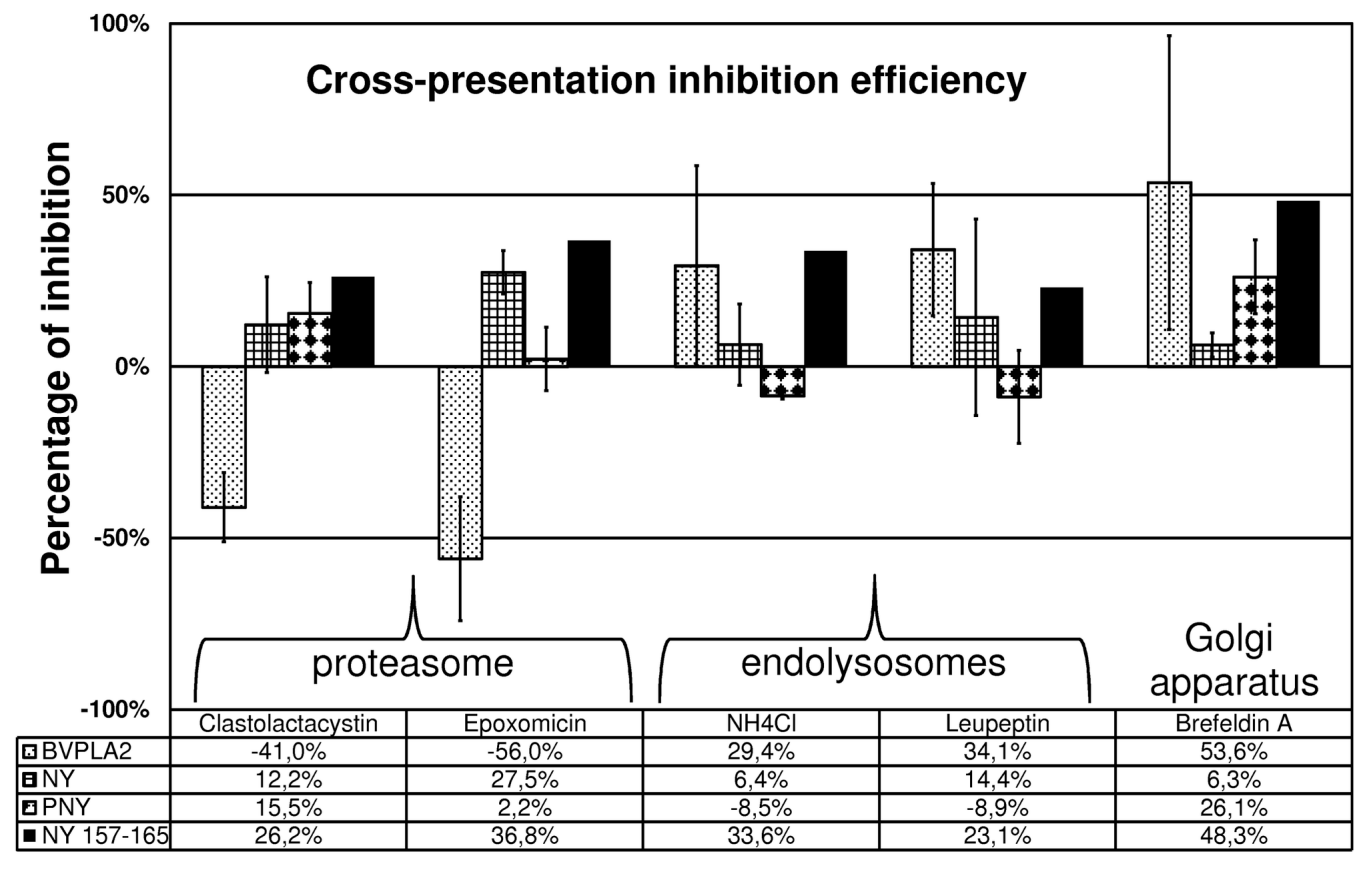
Histogram of percentage inhibition of peptide derived from NY(s) cross-presentation. The results represent mean ± SD of percentage inhibition of at least two independent experiments in duplicate. DCs were pretreated with inhibitors before incubation with proteins or peptide. Four CD8+ T-cell lines from two different healthy donors, generated with PNY- or NY_157-165_-pulsed autologous DCs, were used in an INF-γElispot Assay. bvPLA2m- and PNY-derived peptide cross-presentation was inhibited by brefeldine A; proteasome inhibition increased bvPLA2m cross-presentation; no statistically significant difference was observed between cross-presentation efficiency of NY(s) after DC inhibitor treatment.

Regarding data from a statistical point of view, NY(s) cross-presentation was not significantly affected by the drug treatment. In contrast, the presentation of peptide derived from PNY was inhibited by BFA, a drug disassembling the Golgi apparatus and inhibiting newly synthesized MHC class I molecules. The drug treatment prevented 26% of peptide cross-presentation (p< 0.001) (Figure 5). Unfused bvPLA2m was sensitive to clasto-Lactacystin β-lactone (p= 0.029) and epoxomicin (p= 0.016), two irreversible proteasome inhibitors which enhanced protein cross-presentation up to 50% more, and to BFA, which inhibited at least 50% of the peptide presentation (Figure 5). DCs treated with inhibitors and then pulsed with the peptide, NY_157-165_, were less immunogenic than DCs not treated with inhibitors and pulsed with the same peptide. This result showed that all inhibitors affected the level of HLA molecules at the DC surface. This result has already been described by Rock *et al*. [31] who demonstrated that, by blocking enzyme degradation, inhibitors diminished peptide production by decreasing peptide loading onto the MHC molecules, thus inducing their structural instability.

Altogether, these results suggest that the cross-presentation pathway of PNY is similar to that found for P30-34, and that peptides are presented onto newly synthesized MHC class I molecules.

### Cross-presentation of HLA-A*02-restricted peptide, NY_157-165_, at the surface of T1 cells

**Figure 6:**
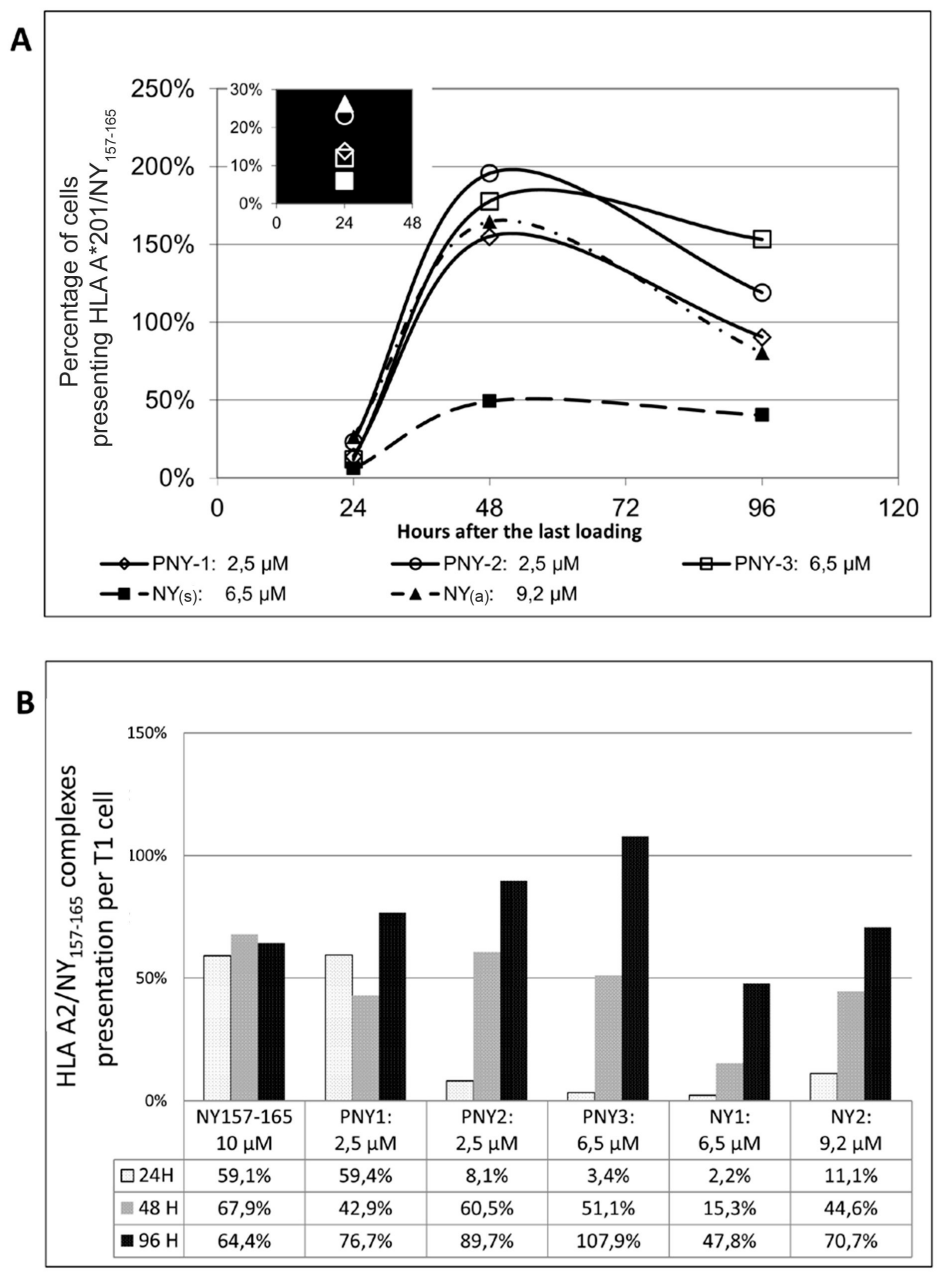
HLA-A*02/NY_157-165_ presentation kinetics. T1 cells were incubated separately with PNY1, PNY2, PNY3, NY(s) and NY(a). NY_157-165_ HLA-A*02/NY_157-165_ complexes were detected with specific antibody and labeling was assessed by flow cytometry. A) Percentage of cells cross-presenting HLA-A*02/NY_157-165_; the results represent the percentage of cells presenting HLA-A*02/NY_157-165_ compared with the day control cells. The day control cells were freshly peptide-pulsed T1 cells. The highest number of cells presenting complexes was reached 48 h after the third loading. B) Percentage of complexes per cell; the number of fluorophores per cell, proportional to the number of complexes per cell, was evaluated with median fluorescence intensity. The results represent the percentage of complexes, calculated from the number of complexes presented by T1 cells loaded at T0 compared with the number of complexes presented by freshly peptide-pulsed T1 cells. The results showed that the number of HLA-A*02/NY_157-165_ complexes per cell increased with time and was higher with PNY-loaded cells.

To evaluate the benefit of bvPLA2m in PNY to promote NY(s) peptide-derived cross-presentation, T1 cells were chosen as they have no calreticulin at their surface, avoiding the NY(s) contribution of PNY binding. To study the processing of NY(s) and its peptide presentation by T1 cells, the high-affinity antibody fragment F(ab) that specifically recognizes the NY_157-165_ peptide/HLA-A*02 complex was used. At first, the optimum protein pulse concentration and lag time after loading T1 cells, to detect NY_157-165_ peptide/HLA-A*02 complexes with antibody were determined. T1 cells were loaded in three steps, without washing in between, at T=0 (1st pulse), T=8h (2nd pulse), T=24h (3rd pulse), with either PNY or NY(s), in serum-free culture medium. After loading, the cells were washed and further incubated for up to 48 h. The results showed that three loadings of PNY were necessary for significant cross-presentation of peptides derived from proteins detected with the antibody (data not shown). For NY(s), peptide cross-presentation started after the second loading was complete (data not shown). In a second step, the same experiment was carried out to measure the MHC/peptide complexes appearing up to 96 h after the last loading. Reproducibility was evaluated using three PNY recombinant fusion proteins referred to as PNY1, PNY2 and PNY3, from three different *E. Coli* preparations. At the same time, the stability of HLA-A*02/NY_157-165_ complexes presented at the T1 cell surface was studied through analysis of their appearance and disappearance during the five-day experiment. Two protein-loaded T1 cell control samples were established (Figure 6). Cells loaded with NY(s) were used to compare the cross-presentation efficiency of the three different PNYs. Cells loaded with NY(a) (aggregated form of NY(s)) were used to check cross-presentation efficiency, as particulate proteins are known to be efficiently cross-presented. NY_157-165_-pulsed T1 cells were prepared to monitor the HLA-A*02/NY_157-165_ complex stability referred to as a stability control. T1 cells freshly pulsed with NY_157-165_ were referred to as day controls. Labeled cell populations were analyzed by fluorescence-activated cell sorting (FACS) and were compared with the labeled day control cells. The counting events analysis in channel FL2 defined the percentage of total cells presenting HLA-A*02/NY_157-165_ complexes. Because the amount of fluorescence detected is proportional to the number of fluorophores per cell, the median fluorescence determined the number of complexes per cell. The percentage of cells presenting HLA-A*02/NY_157-165_ complexes (C) was calculated as:

$$F=\frac{\text{Fl2 counting events of protein-loaded T1 cells}}{\text{Fl2 counting events of day control-pulsed T1 cells}}x100$$

The percentage of complexes per cell (P) was determined according to the following equation:

$$P=\frac{F-f0}{fd-f0}x100$$

With:

F = Median fluorescence of protein-loaded T1 cellsf0 = Median fluorescence of unloaded, labeled T1 cells
fd = Median fluorescence of day control-pulsed T1 cells

The results showed that the number of cells presenting HLA-A*02/NY_157-165_ (C) complexes and the number of complexes per cell (P) increased with time (Figure 6A and 6B). The highest percentage of cells loaded with proteins was reached 48 h after the last loading and represented 155% for PNY1, 196% for PNY2, 178% for PNY3, 49% for NY(s) and 165% for NY(a), of the day-control positive labeled cells (Figure 6A). NY(a)-loaded T1 cells supplied three times more HLA-A*02/NY_157-165_-presenting cells than NY(s)-loaded cells. As expected, NY(a) was successfully cross-presented. The three recombinant proteins, PNY1, PNY2, and PNY3, were cross-presented by up to four times the proportion of cells loaded with NY(s). Consequently, fusion of antigen to bvPLA2m augmented the proportion of antigen-loaded cells able to cross-present.

To check HLA-A*02/NY_157-165_ complex stability, protein-loading cells were compared with NY_157-165_-pulsed cells treated in the same manner as protein-loaded cells and conserved over the five day experiment. Mean fluorescence comparison between fresh and conserved NY_157-165_-pulsed cells showed that from 24 h to 96 h after the last loading, the peptide remained stable at the surface of the T1 cells. A mean of 64% of the complexes detected with freshly peptide-pulsed T1 cells was still detected over the five day experiment (Figure 6B). At the same time, this sample analysis indicated the good inter-assay reproducibility of the experiment, with less than 10% variation (Figure 6B).

PNY3-loaded cells revealed a higher number of complexes at their surface, with twice the number of complexes presented by NY(s)-treated cells at the same loading concentration (6.5 µM) (Figure 6B). NY(a) needed to be concentrated at around 9.2 µM to be cross-presented to the same extent as PNY1 and PNY2, which had a loading concentration of 2.5 µM. Thus, comparison of NY(a) and PNY loading concentrations indicated that four times less protein was required for the same cross-presentation efficiency.

Increasing the PNY loading concentration did not proportionally increase the number of complexes formed at the cell surface. From a PNY loading concentration of 2.5 µM to 6.5 µM, an increase of 20% in the number of complexes was observed, indicating that the maximum intracellular protein processing and presentation capacity was reached (Figure 6A and 6B). To summarize, bvPLA2m increased by four-fold the proportion of loaded cells able to cross-present peptide derived from a whole antigen fused to it, and by two fold the number of HLA A*02/NY_157-165_ complexes presented per cell.

### PNY generated CD8+ T-cell lines which recognized HLA-A*02 melanoma cells

**Figure 7:**
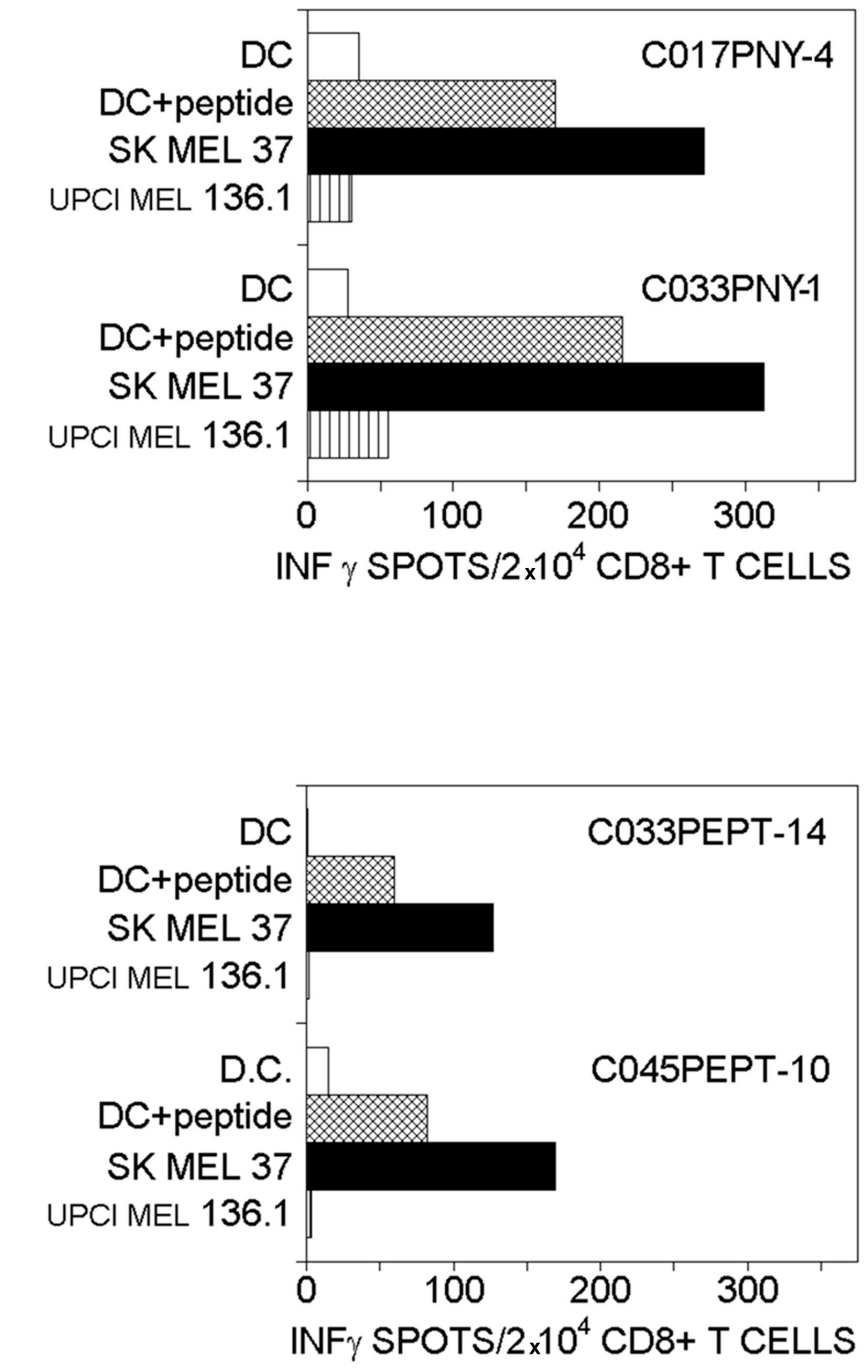
Recognition of SK-MEL 37 melanoma cells expressing NY-ESO-1 antigen and HLA-A*0201 molecules by CD8+ T-cells specific to NY(s). The HLA-A*0201, NY-ESO-1**^-^** melanoma cell line, UPCI-MEL 136.1, was used as a negative control. The CD8+ T-cell line was raised with monocyte-derived HLA-A*02 dendritic cells loaded with either peptide NY_157-165_ or the recombinant protein, PNY. The results are from a unique experiment in singlet.

The NY_157-165_-specific CD8+ T-cell lines induced with peptide-pulsed DCs or DCs loaded with the recombinants proteins, NY(s) or PNY, were tested for their ability to recognize human melanoma cells expressing NY-ESO-1. Two melanoma cell lines were selected: SK-MEL-37 which is HLA-A*0201+ and NY-ESO-1+, and UPCI-MEL-136.1 which is HLA-A*0201+ and NY-ESO-1-. UPCI-MEL 136.1 was used as a negative control. These melanoma cell lines were examined for their abilities to stimulate IFN-γ production by CD8+ T-cells specific for the NY_157-165_ epitope in the context of HLA-A*0201 (Figure 7). Of the seventeen CD8+ T-cell lines generated with the synthetic peptide, NY_157-165_, only two were capable of recognizing SK-MEL-37(C033PEPT-14 and C045PEPT-10) (Figure 7). Of the CD8+ T-cell lines generated with PNY, two were capable of recognizing SK-MEL-37(C017PNY-4 and C033PNY-1) (Figure 7). No CD8+ T-cell line stimulated with NY(s) was able to recognize the melanoma cell line. These results indicated that, of the CD8+ T-cell lines generated with DCs prepared with whole antigen, only 12% carried TcR able to interact with tumor cell MHC/peptide complexes.

## Discussion

Of the available vectors for preparing highly immunogenic and efficient DC cancer vaccines, bvPLA2 was assumed to be a good candidate for antitumor DC-based vaccines as its interaction with the membrane allows antigen internalization by fusion to its C terminus. Here, the mutant bvPLA2m was shown to be able to augment the capture of NY(s) by DCs, enhance NY(s)-derived peptide cross-presentation and promote the cross-priming of NY-ESO-1-specific CD8+ T-cells capable of recognizing human melanoma cells expressing NY-ESO-1.

The bvPLA2m in PNY increases the anchorage of NY(s) to the surface of the DCs at least twice as much as NY(s) alone during the first two hours of incubation, taking into account the fact that NY-ESO-1 is internalized into the DCs via the calreticulin at the surface membrane [22]. This interesting bvPLA2 feature, due to its strong and irreversible membrane interaction [14], will help antigens fused to it to be anchored to the membrane and then to be internalized in greater quantities than antigen alone.

According to the PNY localization microscopy analysis, PNY entered cells via energy-dependent uptake linked to vesicular transport, reached the early endosomes, and was delivered to the late endo-lysosomes. In this latter sub-cellular compartment, PNY was processed into peptides presented on the MHC class II molecules, forming complexes able to induce, *ex vivo*, NY(s) specific to CD4+ T-cells. Of these, the MHC II-restricted peptide-epitope, NY-ESO-1_87-111_-specific CD4+ T-cell was characterized in the context of HLA-DRB3*0101, indicating that bvPLA2m allows NY(s) presentation by the DCs without impairing the processing of the natural cancer/testis antigen, NY-ESO-1. Surprisingly, no bvPLA2m-specific CD4+ T-cells were generated with DCs loaded with PNY. This result was unexpected as confocal microscopic image analysis showed that PNY was localized in late endosomes. Maillère *et al*. demonstrated that proteins containing disulphide bonds were resistant to cathepsin B and D degradation in the endo-lysosome compartment [32]. The protein bvPLA2m contains five disulfide bonds which may lead to resistance to enzyme cutting. Moreover, it can be supposed that the high NY(s) degradation supplies sufficient degradation products to saturate recycled and newly synthesized MHC class II molecules, which are then directed to the membrane surface. During this process, bvPLA2m may then translocate to the cytosol and so definitely escapes acid protease hydrolysis. This hypothesis is supported by the inhibitor experiments, which showed that inhibition of the proteasome affected cross-presentation of the peptide derived from bvPLA2m. This suggested that bvPLA2 may be located in the cytosol, indicating that the protein can escape from the endosome. When the proteasome is inhibited, cross-presentation of peptide derived from bvPLA2 increases significantly. The proteasome is involved in the generation of many MHC class I-restricted epitopes. However, this is not the only cytosolic peptide cleavage system, as tripeptidyl peptidase II (TPPII) acting independently of the proteasome activity, and probably helped by the endoplasmic reticulum aminopeptidase associated with antigen processing (ERAAP), has been shown to play an important role in generating epitope [33]. The source of peptides derived from bvPLA2 could be TPPII cleavage. Because proteasomes can either generate or destroy MHC class I epitopes [34], it seems that proteasome inhibition prevents peptide destruction of bvPLA2. Thus it can be predicted that, in the context of a functional proteasome, only a few MHC class I-restricted epitopes will be presented, rendering bvPLA2 as a low-level immunogen. Actually, *in vitro* stimulation of CD8+ T-cells by DCs loaded with PNY produced only three T-cell lines, out of the seventeen induced, which were specific to NY(s). As cross-presentation efficiency has been suggested to be dependent on the ability of the proteins to access the MHC class I processing machinery in the ER lumen directly, and escape endosomal degradation [35], bvPLA2m might be a vector which can induce efficient antigen cross-presentation.

DCs from HLA-A*02 donors loaded with PNY were capable of inducing, *ex vivo*, CD8+ T-cells specific to NY_157-165_ MHC class I-restricted epitopes, indicating that intracellular PNY traffic leads to the MHC I loading compartment where whole antigen processing generates the same antigenic peptides as are presented at the surface of tumor cells. The immunogenicity of each protein (NY(s) and PNY)-loaded dendritic cell vaccine, was studied through their ability to prime NY-ESO-1-specific CD8+ T-cells. Considering that each cell line was derived from one CD8+ T-cell, the mean naive NY_157-165_ precursor frequency was at least 1.1 10^-6^± 0.65 10^-6^, close to that measured in the peripheral blood of healthy, seronegative donors [36]. Moreover, 75% of the CD8+ T-cell lines generated with PNY- and NY(s)-loaded DCs are NY_157-165_-specific, confirming the immune dominance of the epitope. PNY-loaded DCs induced CD8+ T-cell lines as much as NY_157-165_-pulsed DCs and half as much as NY(s)-loaded DCs. Thus, vaccine prepared with PNY-loaded DCs seems to be more immunogenic than vaccine prepared with NY(s)-loaded DCs. However, dose–response experiments, in which DCs previously pulsed with various concentrations of protein or peptide were exposed to PNY and NY(s)-specific CD8+ T-cells, did not show any significant difference between the delivery systems.

The cross-presentation kinetics of peptides derived from NY(s) were analyzed using an antibody that recognizes the NY_157-165_/HLA-A*02 complexes. The results showed that the level of NY(s)-derived peptides cross-presented per cell gradually increased over time. The maximum number of NY_157-165_ peptide/HLA-A*02 complex-presenting cells was attained 48 h after loading, whatever protein delivery system was used. At that time, the difference between NY(s) and PNY peptide-derived presenting cells was significant and the proportion of NY_157-165_-presenting cells was four times higher with PNY-loaded T1 cells than with NY-loaded T1 cells. From two to four days after the last loading, PNY-loaded T1 cells presented twice as many HLA A*02/NY_157-165_ complexes per cell as soluble NY(s)-loaded T1 cells, whereas 24 h later there was no difference between them. This explains why there was a difference in the ability of NY(s)-and PNY-loaded DCs to generate NY-specific CD8+ T-cells whereas no difference was observed in their abilities to activate T-cells. Experimentally, CD8+ T-cells had to be co-cultured with loaded DCs for weeks, renewed weekly, in order to be generated. On the other hand, CD8+ T-cells were co-incubated for only 24 h to be activated and 24 h is not sufficient for PNY to be efficiently cross-presented. Hence, compared with receptor-mediated endocytosis, bvPLA2m increases the anchorage of antigens two-fold and enhances antigen immunogenicity as much as a specific peptide. Moreover, vaccines prepared with whole-antigen-loaded APC, have the advantage of producing a broad peptide epitope library.

Compared with NY(s) presentation by T1 cells, without calreticulin at the surface of the membrane, PNY doubles the number of HLA A*02/NY_157-165_ complexes formed at the cell surface and augments the number of cells able to cross-present these complexes by four-fold. In contrast to peptides derived from NY(s), peptides derived from PNY, specific to NY(s), are presented on nascent MHC class I molecules.

Of the specific NY _157-165-_CD8+ T-cell lines generated with either peptide-pulsed DCs or whole-antigen-loaded DCs, only 12% were capable of recognizing the SK-MEL37 tumor-cell line. This result illustrates recent vaccination trial data assessing the immunogenicity of an NY-ESO-1 vaccine [37].

Finally, antigens fused to bvPLA2m seem to be first accumulated in cells, in particular in late endosomes, before translocation into the cytosol and peptide cross-presentation. This process is estimated to take 48 h, which is sufficient for cross-presenting DCs to migrate towards the lymph node and activate antigen-specific T-cells [38]. Thus, bvPLA2m ensures continuous peptide loading by prolonging cross-presentation with as much antigen as is available in APC. Furthermore, bvPLA2m augments by at least eight times the cross-presentation of peptide derived from the antigen and shows low immunogenicity, which is critical for vaccine development.

## Materials and Methods

### Culture media, cytokines, reagents, peptides and cell lines

Oligonucleotides were obtained from Eurobio (Les Ulis, France). AIM-V and IMDM culture media were purchased from Gibco Invitrogen. IMDM was supplemented with 10% human AB serum (Biowest, AbCys, Paris, France), 0.24 mM aspartate, 0.55 mM arginine, 1.5 mM glutamine (Sigma) and 1% v/v penicillin–streptomycin (Gibco Invitrogen). Cytokines were obtained from R&D, except for IL-15, which was obtained from Peprotech and rhIL4 from TEBU, Le Perray en Yveline, France. Human albumin was from LFB (Courtaboeuf, France). Conjugates for flow cytometry were obtained from BD Biosciences. Peptide NY_157-165_ was obtained from Neosystem (Strasbourg, France). Fluorochrom Alexa-Fluor-488 succinimidyl ester and Alexa-Fluor-488 maleimide were purchased from Molecular Probes. The melanoma cell lines SK-MEL37 (HLA-A*0201^+^ NY-ESO-1^+^) [39,40] and UPCI-MEL 136.1 (HLA-A*0201^+^, NY-ESO-1^-^) [30,41] were a kind gift from Dr. Hassan Zarour. The T1 cell line was obtained from ATCC (Manassas VA). The HLA A*02 /NY_157-165_ antibody was a kind gift from Dr. Held Gerard.

### Recombinant proteins, bvPLA2H34Q and NY-ESO-1, and the fusion protein, bvPLA2H34Q-NY-ESO-1

#### bvPLA2H34Q (bvPLA2m)

Expression and purification of the protein has been described previously [42].

#### NY-ESO-1 (NY(s))

The NY-ESO-1 coding sequence from plasmid pcDNA 3.1 (-) (a kind gift from Dr. Hassan Zarour) was cleaved with the two restriction enzymes, *BamH* I and *Hind* III. The NY-ESO-1 coding sequence was subcloned into the pQE 80 vector (Qiagen) at the *BamH* I and *Hind* III restriction sites. Expression of the protein in the *E. Coli* Rosetta strain (Novagen), extraction of the inclusion bodies and His-tag-mediated purification were performed using the procedure used for bvPLA2m. Protein refolding was performed by dialysis against a 50 mM Tris; 10% glycerol; 50 mM NaCl; 5 mM reduced glutathione; 1 mM oxidized glutathione; pH 10 buffer. The final product yield was 2 mg of 95% pure protein per liter of culture. The protein sequence was verified by N-terminal protein sequencing. NY(s) in its native state was conjugated with an Alexa-Fluor-488 carboxylic acid succinimidyl ester following the manufacturer’s instructions. Denatured NY(s), referred to as NY(d), was prepared with a 2 M urea buffer containing 1 mM TCEP and was labeled with Alexa Fluor®488 maleimide according to the manufacturer’s procedure.

#### Aggregated NY-ESO-1 (NY(a))

Urea from denatured NY(s) was eliminated by successive dialysis against a 50 mM Tris; pH 10 buffer. Aggregation was assessed using a dynamic light scattering experiment, measuring the hydrodynamic diameter of the proteins.

#### PLA2H34Q-NY-ESO-1 (PNY)

NY-ESO-1 coding sequence was amplified by PCR and introduced into the pQE-PLA2H34Q expression vector at the *BamH* I and *Hind* III restriction sites. The oligonucleotides used for PCR inserted a flexible linker at the N-terminus of the protein, after the *BamH* I restriction site. The resulting protein contained the sequence MRGSHHHHHHSPFR, the bvPLA2m coding sequence, the linker GSGGSGGSG, and the NY-ESO-1 coding sequence. Protein expression was achieved in *E. Coli* Rosetta strain inclusion bodies. The fusion protein was isolated as for recombinant bvPLA2m. It was refolded by successive dialysis against a 10 mM EDTA; 100 mM Tris pH 10; 10% glycerol; 8 mM cysteine and 1 mM cystine buffer at decreasing guanidine hydrochloride concentrations (2 M, 1.5 M, 0.9 M, 0.5 M and none). The last step in folding was performed in PBS. PNY(d), the denatured form of PNY, used for assessing protein anchorage to the DC membrane, was prepared with PNY in a 2 M urea; 1 mM TCEP buffer and labeled with Alexa-Fluor®488 maleimide.

#### bvPLA2H34Q-N9V-M9V (P30-34)

As described by the Babon *et al*. study, this is a recombinant fusion protein composed of two HLA-A*02-restricted epitopes from cytomegalovirus pp65, N9V and M9V, fused to bvPLA2H34Q. The protein was also produced in *E. Coli* as previously described [16].

### Circular dichroism (CD) spectroscopy

CD experiments were performed on a CD6 spectrodichrograph (Jobin-Yvon Instruments). The measurements were made at 22°C and 37°C with constant N2 flushing, as described previously [43]. Far-UV spectra were measured in 0.5 mm- and 10 mm-path-length quartz cells. The samples were buffered using 10 mM sodium phosphate (pH 8) at a concentration of 10 µM. Scans were recorded using a bandwidth of 2 nm and an integration time of 1 s at a scan rate of 0.5 nm/s. Each far-UV spectrum represents the average of 20 scans. The spectra were corrected against the blank, and then a smoothing algorithm was applied with the minimum filter in the CD6 software (CDMax, filter 5). The mean residue ellipticity in far-UV [θ] was calculated from the relationships [θ]F=(100×θm)/(C×l×N) and [θ]N=(100×θm)/(C×l), where θm is the measured ellipticity in degrees, C is the concentration in moles per liter, l is the cell path length in centimeters and N is the number of residues. The value, 100, arises from converting the concentration in moles per liter to decimoles per cubic centimeter. The mean residue ellipticity [θ]F and molar ellipticity [θ]N units are expressed in degrees per square centimeter per decimole residue (deg cm^2^ /dmol res) and in degrees per square centimeter per decimole (deg cm^2^ /dmol), respectively [44].

### Dynamic light scattering (DLS)

Each of the study proteins was characterized as being in a monomer or aggregate state using light scattering. Hydrodynamic diameters were measured at 25°C using a Malvern Nanosizer ZS instrument equipped with a 4 mW He–Ne solid-state laser operating at 633 nm. Backscattered light was detected at 173°, and the mean protein diameter was calculated from the quadratic fitting of the correlation function over 15 runs of 10 s. All measurements were performed in triplicate on proteins diluted to 0.5 mg/ml in phosphate-buffered saline (PBS). The data were collected and analyzed using the Stokes-Einstein equation.

### Generation of immature monocyte-derived dendritic cells

Peripheral blood mononuclear cells (PBMC) were prepared from blood cells collected at the Etablissement Français du Sang (EFS, Rungis, France), as buffy-coat preparations from anonymous healthy donors who gave informed consent, in accordance with EFS guidelines. These PBMCs were isolated by density centrifugation on Ficoll-paque^TM^ gradients (Pharmacia, Amersham biotechnologies) and were used to prepare immature DCs as described in [16] with minor modifications. Adherent cells were cultured in AIM-V medium supplemented with 1000 units/mL rhGM-CSF and 1000 units/mL rhIL-4. Five days later, the immature DCs were harvested, centrifuged, frozen, or used to stimulate autologous T-cells. The CD4+ or CD8+ T-stimulator cells were resuspended in AIM-V, at 10^6^/mL supplemented with peptide (10 µM) or protein (3 µM) and 1 µg/mL LPS. The cells were incubated for 1.5 h at 37°C. DCs pulsed with peptide or protein were then washed and resuspended in IMDM. In order to stimulate CD8+ T-cells, LPS was replaced with Ribomunyl® (Laboratoire Pierre Fabre Médicament, Castres, France) and the culture medium was supplemented with 5 µg/mL human β2-microglobulin.

### Protein anchorage to the DC surface

As for NY(s), PNY in its native form, and following denaturation, was labeled with the Alexa Fluor®488 fluorophore according to the manufacturer’s procedure. The fusion protein, P30-34, used in the Babon *et al*. study was also conjugated with Alexa Fluor®488. The degree of labeling was calculated according to the manufacturer’s procedure.

### Internalization assay using confocal microscopy

Immature DCs were suspended at 10^6^ cells/mL in AIM-V. To localize PNY into early endosomes, DCs were pre-incubated with 60 µg/mL transferrin-Texas Red for 30 min, washed and then incubated with 3 µM PNY-Alexa-Fluor®488 for 30 min. To follow the fluid phase entry, DCs were incubated simultaneously with 1 mg/mL 40 kDa dextran-Texas Red and 3 µM PNY-Alexa Fluor®488 at the time indicated in Figure 2A. Immature DCs were pre-treated for 2 hours with cytochalasin B, before incubation for 2 hours with co-localization marker, dextran or transferrin (red) and PNY-Alexa-Fluor®488 (green). DCs were then incubated for 2 hours at 4°C with PNY-Alexa-Fluor®488 and 40 kDa dextran-Texas Red. For microscopy analysis, the DCs were washed twice with PBS, fixed with 3% paraformaldehyde and spotted onto a slide by centrifugation with a cytospin. The cells were then mounted with fluoromount and covered with a coverslip. Cells were observed under a NIKON confocal microscope. A snapshot was taken every 0.4 µm using a 60x objective. Medial optical sections were selected to analyze the intracellular localization of PNY-Alexa-Fluor®488.

### CD4+ T-Cell induction

Autologous CD4+ T-cells were positively isolated from PBMCs with immunomagnetic beads (Miltenyi Biotech). 10^5^ T-cells were added to 10^4^ DCs loaded using the recombinant protein in 0.2 mL culture medium per well with 1000 units/mL IL-6 and 10 ng/mL IL-12. On day 7, and weekly thereafter, the lymphocytes were restimulated with autologous DCs pulsed with peptide or protein in a culture medium supplemented with 10 units/mL IL-2 and 5 ng/mL IL-7. The stimulated CD4+ T-cells were analyzed for their specificity for autologous DCs, either loaded with 3 µM PNY, NY(s) or bvPLA2m, or pulsed with 10 µM NY_87-111_, in interferon-gamma (IFN-γ) ELISPOT assays at day 21.

### CD8+ T-cell induction

Autologous CD8+ T-cells were positively isolated from PBMCs using immunomagnetic beads (Miltenyi Biotech) and added (1.5×10^5^) to 3×10^4^ DCs pulsed with peptide or recombinant protein in 0.2 mL culture medium per well with 1000 units/mL IL-6 and 5 ng/mL IL-12. On day 7, the lymphocytes were restimulated with autologous DCs pulsed with peptide (10 µM) or protein (3 µM) in a culture medium supplemented with 10 units/mL interleukin 2 (IL-2) and 5 ng/mL interleukin 7 (IL-7). On day 14, and weekly thereafter, the lymphocytes were restimulated in the same way in the presence of 25 ng/mL IL-7, 25 ng/mL IL-15, 500 U/mL IFN-γ and 1 µg Ribomunyl. These stimulated CD8+ T-cells were analyzed for their specificity for autologous DCs, either loaded with 3 µM PNY or NY(s), or pulsed with 10 µM NY_157-165_, in IFN-γ ELISPOT assays at day 21. T-cell lines were considered to be antigen-specific when their response was expressed by more than 60 IFN-γ spots and at least twice the number of spots obtained with unloaded DCs. Cells from four healthy donors were used. A series of 10 to 20 wells per antigen (NY(s), PNY, NY_157-165_), and per donor, were seeded.

### Assessment of T-cell responses to peptide, proteins and tumor cells

The recognition of APCs pulsed with peptide, proteins and tumor cells, was assessed using ELISPOT assays specific for hIFN-γ. For the ELISPOT assays, multiscreen HA plates (Millipore) were coated with 10 µg/mL monoclonal antibody (mAb) 1-D1K anti-hIFN-γ(Mabtech) in PBS (Gibco Invitrogen), overnight at 4°C. Unbound mAb was removed by four washes with PBS. The plates were then blocked with IMDM-10% human serum for 1 h at 37°C. Between 5×10^3^ and 20×10^3^ CD4+ T-cells per well were seeded in the mAb coated plates. DCs loaded with peptide or proteins (5×10^3^) were added. To evaluate the CD8+ T-cell response, between 10^4^ and 4.5×10^4^ CD8+ T-cells per well were added to 5×10^3^ DCs loaded with peptide or proteins or to 20×10^3^ melanoma cells. Control wells contained T-cells, either alone or with unloaded DCs or tumor cells alone. The culture medium was IMDM at a final volume of 0.2 mL/well. After incubation at 37°C in 5% CO_2_ atmosphere for 20 h, the cells were removed by washes with PBS-0.05% Tween 20. Captured hIFN-γ was detected by incubation for 2 h at room temperature with biotinylated mAb anti-hIFN-γ, 7-B6–1 (Mabtech) in PBS-0.5% BSA. The plates were washed four times with PBS-Tween, and extravidin-alkaline phosphatase conjugate (Sigma), diluted 1:4000, was added for 1 h at room temperature. Unbound complex was removed by three washes with PBS-Tween and three washes with PBS alone. Alkaline phosphatase staining was performed with NBT/BCIP (Sigma) for 7 min and stopped by rinsing the plates under running tap water. Spot numbers and sizes were determined with an AID ELISPOT reader (Straβberg, Germany).

### Titration of CD4+ and CD8+ T-cell reactivity

#### Titration of CD4+ T-cells

To titrate NY(s)-and PNY-specific CD4+ T-cell reactivity, NY(s) and PNY cells presenting autologous DCs, pulsed with different concentrations ranging from 3 nM to 3 µM of either NY(s) or PNY, were used to stimulate specific CD4+ T-cells in a 20-h IFN-γ ELISPOT.

#### Titration of CD8+ T-cells

To compare the efficiency of NY(s) and PNY cross-presentation, autologous DCs were pulsed with different concentrations of NY or PNY proteins ranging from 2 nM to 2 µM, or synthetic peptide NY_157-165_ with concentrations ranging from 20nM to 20 µM. The loaded DCs were then used to stimulate NY- or PNY-specific T-cell lines in a 20-h IFN-γ ELISPOT assay.

### Inhibitor experiments for characterization of the MHC class I molecule loading compartment

For experiments with inhibitors, DCs were treated with the inhibitor 45 min before and during the antigen pulse, then washed and cultured with the same doses of inhibitor for 4 h to allow eventual processing prior to incubation with the T-cells on the ELISPOT plates. After treatment with NH_4_Cl (50mM), leupeptin (10 µM) and Brefeldin A (BFA, 10µM), DCs were fixed with 0.005% glutaraldehyde before incubation with the T-cells and each treatment was tested in duplicate. However, because the effect of BFA is reversible, half of the BFA-treated DCs were washed without glutaraldehyde fixation before incubation with T-cells. These recovered DCs were used as the BFA treatment control for each loaded DC sample treated with BFA.

### Cross-presentation of the HLA-A*02-restricted-peptide, NY_157-165_

T1 cells were incubated either with the recombinant protein NY(s) or the fusion protein PNY at the concentration indicated in the results section, or pulsed with 20 µM NY_157-165_ peptide. The cells were loaded three times, once every 8 h. Controls were used to compare the results obtained from protein-loaded T1 cells which included two control samples. The first sample was prepared with T1 cells pulsed three times with 20 µM peptide NY_157-165_, as the DCs were loaded with proteins. This sample is referred to as a stability control, which indicated peptide stability at the T1 cell surfaces over the experimental time. The second control determined the level of HLA-A*02 molecules associated with the NY_157-165_ T1 cell surface. This sample was renewed for every experiment and was composed of T1 cells freshly pulsed with 20 µM NY_157-165_ for 2 h, washed, fixed, labeled and immediately analyzed by flow cytometry. This internal control sample is referred to as the day control. HLA-A*02 /NY_157-165_ complexes were labeled 24, 48 and 96 h after the last loading. A fraction of 50 000 cells was analyzed by flow cytometry (FACS). HLA-A*02/NY_157-165_ complexes were detected with antibodies against HLA-A*02 /NY_157-165_, as described previously [23]. Briefly, the loaded cells were harvested, washed twice with the culture medium without serum and once with PBS. The cells were then labeled with the first antibody fragment F(ab) (a kind gift from G. Held) diluted to 20 µg/ml in PBS-10% SVF for 2 h at room temperature to detect HLA-A*02 /NY_157-165_. The second stage of cell labeling included two washes in PBS, and the first antibody detection with goat anti-mouse whole immunoglobulin G polyclonal antibody conjugated to phycoerythrin at 20 µg/ml, from BD Bioscience (Rungis, France). After the last two washes in PBS, the labeled cells were analyzed by flow cytometry. Unloaded cells labeled with antibodies were used to establish a negative control and to set the fluorescence base-line. Labeling efficiency was checked with the day control sample.

### Statistical analysis

One-way analysis of variance (one-way ANOVA) was used to test for statistically significant differences within each drug treatment group (clasto-Lactacystin β-lactone, epoxomicin, NH_4_Cl, leupeptin, BFA) and within each protein group (NY(s), PNY, bvPLA2, NY_157-165_). The one-way ANOVA test was applied to raw data. Analyses were performed with SigmaStat. Two independent experiments, each performed in duplicate, were analyzed for each protein cross-presentation inhibition. This statistical test required a normal distribution of data, equal variance and a confidence coefficient p <0.05.
